# Rationale and design of the *CAPAMIS *study: Effectiveness of pneumococcal vaccination against community-acquired pneumonia, acute myocardial infarction and stroke

**DOI:** 10.1186/1471-2458-10-25

**Published:** 2010-01-19

**Authors:** Angel Vila-Corcoles, Inmaculada Hospital-Guardiola, Olga Ochoa-Gondar, Cinta de Diego, Elisabet Salsench, Xavier Raga, Cruz M Fuentes-Bellido

**Affiliations:** 1 Primary Care Service of Tarragona-Valls, Institut Catalá de la Salut, Tarragona, Spain; 2 Department of Microbiology and Laboratory, Hospital Santa Tecla, Tarragona, Spain; 3 Department of Medicine and Surgery, Faculty of Medicine and Health Sciences, Rovira i Virgili University, Tarragona, Spain

## Abstract

**Background:**

The 23-valent polysaccharide pneumococcal vaccine (PPV-23) is recommended for elderly and high-risk people, although its effectiveness is controversial. Some studies have reported an increasing risk of acute vascular events among patients with pneumonia, and a recent case-control study has reported a reduction in the risk of myocardial infarction among patients vaccinated with PPV-23. Given that animal experiments have shown that pneumococcal vaccination reduces the extent of atherosclerotic lesions, it has been hypothesized that PPV-23 could protect against acute vascular events by an indirect effect preventing pneumonia or by a direct effect on oxidized low-density lipoproteins. The main objective of this study is to evaluate the clinical effectiveness of PPV-23 in reducing the risk of pneumonia and acute vascular events (related or nonrelated with prior pneumonia) in the general population over 60 years.

**Methods/Design:**

Cohort study including 27,000 individuals 60 years or older assigned to nine Primary Care Centers in the region of Tarragona, Spain. According to the reception of PPV-23 before the start of the study, the study population will be divided into vaccinated and nonvaccinated groups, which will be followed during a consecutive 30-month period. Primary Care and Hospitals discharge databases will initially be used to identify study events (community-acquired pneumonia, hospitalisation for acute myocardial infarction and stroke), but all cases will be further validated by checking clinical records. Multivariable Cox regression analyses estimating hazard ratios (adjusted for age, sex and comorbidities) will be used to estimate vaccine effectiveness.

**Discussion:**

The results of the study will contribute to clarify the controversial effect of the PPV-23 in preventing community-acquired pneumonia and they will be critical in determining the posible role of pneumococcal vaccination in cardiovascular prevention.

## Background

### Epidemiology of pneumococcal infections

Infections caused by *Streptococcus pneumoniae *remain a major public health problem throughout the world. Susceptibility to pneumococcal infections varies with age, being highest in young children and older adults, who experience substantial morbidity and mortality (especially in those with underlying high-risk medical conditions) [1,2].

The main reservoir of pneumococcus is the nasopharynx, and the possible outcomes after colonisation are the clearance by the organism, the asymptomatic persistence for several months (carrier state), or the progression to disease. During the disease the bacteria can spread to adjacent mucosal tissues causing mucosal infections (otitis, sinusitis, bronchitis and pneumonias) or it can invade the bloodstream, or other sterile sites, producing an invasive pneumococcal disease (IPD). In adults, *S. pneumoniae *is the most frequent isolate from clinical samples of respiratory tract infections. It is responsible for approximately 50% of overall cases of community-acquired pneumonia (CAP) in different populations [3-5].

### Pneumococcal vaccination

In the last decades, the need for preventive strategies has driven multiple efforts to develop an effective vaccine, however the existence of more than 90 distinct pneumococcal serotypes has largely complicated the development and evaluation of anti-pneumococcal vaccines. To date, only a 23-valent polysaccharide pneumococcal vaccine (PPV-23) for use in adults and a 7-valent conjugate vaccine for use in infants are available in clinical practice [6-9].

The PPV-23 is generally recommended for high-risk and elderly individuals, but its effectiveness (despite numerous meta-analyses) remains controversial [10-19]. It has been demonstrated that PPV-23 provides considerable protection against invasive pneumococcal disease, while a possible protective effect against pneumonia is discussed [20]. The latest Cochrane Systematic Review supports the use of PPV to prevent IPD in adults, particularly in healthy adults, but it concluded that the meta-analysis does not provide compelling evidence to support the routine use of PPV to prevent pneumonia [18].

### Epidemiological link between pneumonia and acute vascular events

For several decades, there has been data that chronic inflamation can promote atherosclerotic disease,[21] and in recent years some studies have also found data supporting the fact that acute infections (especially influenza and pneumonia) are associated with a transient increasing risk of acute vascular events [22-25]. In fact, it has been reported that a combination of the diagnosis of pneumonia and myocardial infarction is relatively common among patients hospitalised with severe pneumonia [24,25]. Thus, given this data, it has been suggested that pneumococcal vaccination could perhaps protect patients from cardiovascular events [26-28].

Following this, a recent case-control study conducted in Canada has reported a 47% reduction in the risk of acute myocardial infarction (AMI) among subjects who had received the PPV-23, however, the posible cardioprotective effect of pneumococcal vaccination has not still been proved [26].

Theoretically, pneumococcal vaccination could protect against acute cardiovascular events by an indirect effect preventing pneumonia (as the latter has been shown to trigger myocardial infarction), although a direct effect of pneumococcal vaccination on oxidized low- density lipoproteins can not be excluded. In fact, animal experiments have shown that pneumococcal vaccination reduces the extent of atherosclerotic lesions and it has been hypothesized that antibodies directed against Streptococcus pneumoniae would also recognize oxidized low-density lipoprotein and impede the formation of foam cells [29].

The posible effectiveness of PPV-23 to prevent myocardial infarction, if it exists, probably has been overestimated in the aforementioned Canadian report; however, given the prevalence of heart disease worldwide, even a modest protective effect (epidemiologically plausible) would translate into a considerable benefit at the population level [30]. Thus, prospective validations (which will determine whether the cardioprotective effect of vaccination is real and better estimate its true protective efficacy) are greatly needed.

### Use of pneumococcal vaccine in the study area

In Catalonia, a region in the northeast of Spain with a population of seven million people, a publicly funded anti-pneumococcal vaccination programme for all individuals 65 years or older (with or without risk factors) and younger individuals with certain high-risk conditions (basically immunodeficiency, chronic heart or pulmonary disease, diabetes mellitus, severe liver disease or chronic nephropathy) began in October 1999. Further, it was extended for all people 60 years or older in 2002. Since then, a free PPV-23 has been offered when the patients came to the primary care services during the annual influenza vaccination campaigns or in any other visit throughout the year. Some years later, vaccination uptake reached approximately fifty percent,[31] and this provided an excellent opportunity to evaluate the vaccine's effects among the population. The potential effects of vaccination in preventing IPD and pneumonia have been analyzed in our reference population in two prior studies [32,33].

This project proposes a large population-based cohort study with the major aim of evaluating the clinical effectiveness of the PPV-23 against CAP as well as evaluating its potential role in cardiovascular prevention among the general population over 60 years.

## Objectives of the capamis study

The primary objective of the planned study entitled ***CAPAMIS ***is to evaluate the clinical effectiveness of the PPV-23 in the prevention of a first episode of **C**ommunity-**A**cquired **P**neumonia, **A**cute **M**yocardial **I**nfarction and **S**troke among the general population over 60 years. Furthermore, the study will evaluate the effectiveness of PPV-23 in reducing the risk of secondary acute vascular events related with prior pneumonia.

Additional objectives include the evaluation of vaccination effectiveness against severity outcomes (including death, need of ICU admission or length of stay in hospital).

Furthermore, a detailed analysis on population-based data on incidence of myocardial infarction and stroke will be performed in different subsets of the population with certain underlying conditions in order to establish, if it exists, the magnitude of vaccination effectiveness in primary and/or secondary cardiovascular prevention.

In addition to these objectives, the clinical effectiveness of revaccination against different outcomes will be evaluated in the subset of participants who have received more than a dose of PPV-23 prior to the study start or during the study period.

## Methods and Design

### Design, setting and study population

Population-based cohort study focused on adults over 60 years in the region of Tarragona, a mixed residential-industrial urban area in the Mediterranean coast of Catalonia, Spain.

Study subjects include 27,000 community-dwelling adults aged 60 years or more assigned to 9 participating Primary Care Centres (PCCs) in the study area, who will followed during a 30-month consecutive period. Six of the nine participating PCCs are located in Tarragona city (PCC Bonavista, PCC Torreforta, PCC Jaume I, PCC Sant Pere i Sant Pau, PCC Tarraco, PCC Sant Salvador) and three PCCs are located in nearby (less than 12 kilometres from Tarragona) smaller municipalities (PCC Salou, PCC Constanti and PCC Morell). Two reference hospitals for the study population are Hospital Joan XXIII and Hospital Santa Tecla, both located in Tarragona city (which has an overall population of approximately 120,000 all-age inhabitants).

It must be noted that, in Spain, primary care physicians are the first point of call for secondary and tertiary care and all citizens are registered in one PCC.

**Figure 1:**
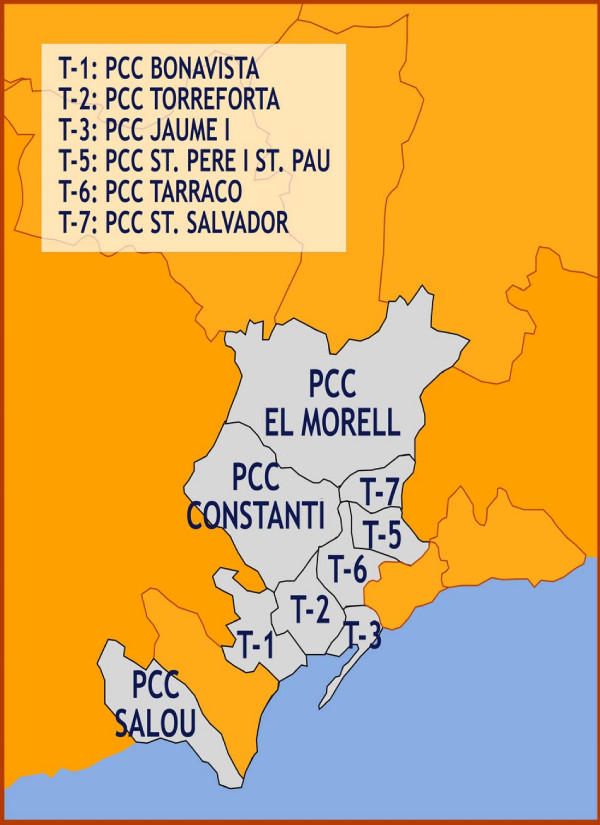
**Research areas of the CAPAMIS Study**. This figure illustrates the geographical distribution of the nine participating Primary Health Care Centers in the region of Tarragona (a mixed residential-industrial urban area, which is located 95 Km from Barcelona, in the mediterranean coast of Catalonia, Spain).

Inclusion criteria for the CAPAMIS cohort include: a) male or female adults aged 60 years or older as of January 1, 2009; and b) registered in any of the nine PCCs participating at study start. Exclusion criteria include residence in a long-term care facility. Subjects will be identified from primary care information systems. Figure 1 shows the study region and illustrates the location of participating centres.

Cohort members will be followed from the beginning of the study (1 January 2009) until occurrence of the first event, the enrolment from the PCC ceases, death, or until the end of the study (30 June 2011).

### Rationale for sample size

The annual incidence of CAP in older adults in the study area is estimated at approximately 10 per 1000 person-year based on prior population-based studies among elderly people in the same study area [34,35]. Incidences of AMI and stroke are estimated in 8 and 12 cases per 1000, respectively, in Spanish population [36,37]. Thus, considering a pneumococcal vaccine coverage of approximately 50%,[31] with a p = 0.05 (two-tailed) the size of the study cohort (N = 27,000 individuals followed during a 30-month period) has an statistical power of 80% to detect a posible vaccination effectiveness of 20% against CAP, a 23% against AMI, a 18% against stroke and a 14% against the combined end-point AMI/stroke.

### Data sources

All participating PCCs have a computerized clinical record system that includes administrative data, medical conditions, prescriptions, laboratory results and registries associated with outpatient visits and diagnoses coded according to the International Classification of Diseases, 10th Revision (ICD-10). This electronic clinical record system will be used to classify vaccination status as well as to identify comorbidities or underlying conditions in cohort members and establish baseline characteristics of the cohort at study start.

During the study period, the primary care electronic registries of the nine participating PCCs will be used as a data source to identify those subjects who have suffered any study event that did not require hospitalisation and were treated as an outpatient during the 30-month study period.

Emergence and hospital diagnosis discharge databases of two reference hospitals (coded according to ICD-9-CM codes (International Classification of Diseases, 9th Revision, Clinical Modification) will be used to identify those cohort members who were admitted to hospital for any study event during the 30-month study period.

A structured data collection instrument will be used to review primary care and hospital clinical records to validate the diagnosis of all cases initially identified by ICD-10 or ICD-9 discharge codes.

### Main outcomes and definitions

The primary endpoints of the *CAPAMIS Study *will be the ocurrence of CAP (hospitalised or outpatient), hospitalisation for AMI, stroke and death from any cause. Table 1 shows definitions and criteria used in collecting primary and secondary outcomes.

**Table 1:** Definitions and criteria used to identify and classify the different endpoints in the *CAPAMIS *Study.

OUTCOMES	DEFINITIONS
A. Community-acquired pneumonia (CAP)*	Pneumonia will be considered when a new radiological infiltrate is identified in a patient with one major criterion (cough, expectoration or fever) or two minor criteria (dyspnea, pleuritic pain, altered mental status, pulmonary consolidation on auscultation and leukocytosis).

**A1. Hospitalised CAP**	Will be considered those pneumonia cases identified on the basis of the listed primary diagnosis codes in hospital discharge databases (ICD-9-CM codes for pneumonia: 480 to 487.0).

**A2. Outpatient CAP**	Will be considered those CAP cases non-hospitalised, and they will be identified from primary care or emergency visits (not hospitalised) with a code registered for pneumonia in the Emergency Unit discharge codes databases (ICD-9-CM codes: 480 to 487.0) or Primary Care Centers diagnosis database (ICD-10 codes: J10.9, J11.9 and J12 to J18).

B. Pneumococcal CAP**	Defined as a patient with CAP from whom Streptococcus Pneumoniae is identified by blood culture, sputum culture or urinary antigen.

**B1. Bacteremic pneumococcal CAP**	Will be considered when S. pneumoniae was isolated from blood specimens or other sterile sites.

**B2. Nonbacteremic pneumococcal CAP**	Will be considered when the patients have a typical clinical syndrome of pneumonia without bacteremia (negative or not performed blood culture), but they have a sputum culture that yielded pneumococci with no other likely bacterial pathogens and/or they have positive Binax-NOW Streptococcus pneumoniae urinary antigen test.

**C. Acute myocardial infarction (AMI)**	Defined as a patient hospitalised with diagnosis of acute or recurrent episode of myocardial infarction. Cases will be identified on the basis of the primary and secondary listed diagnosis codes in the hospital discharge database (ICD-9-CM code for AMI: 410). All initially identified cases of AMI will be further validated by checking hospital medical records, and each case will be classified as related or nonrelated with a prior episode of CAP.

**C1. CAP-related episode of AMI**	Defined as AMI ocurring within the first 30 days after the onset of an episode of CAP.

**C2. CAP-nonrelated episode of AMI**	Case of AMI ocurring more than 30 days after an episode of CAP or in a patient without history of prior CAP.

**D. Hospitalisation for stroke**	Defined as a patient admitted to hospital with a diagnosis of stroke, ictus or cerebral infarction. Cases will be identified on the basis of the primary and secondary listed diagnosis codes in the hospital discharge databases (ICD-9-CM codes for stroke: 430 to 437). Haemorragic strokes (codes 430 to 432) and ischaemic strokes will be considered (codes 433, 434, 436 or 437), but transient ischaemic attacks (code 435) will be excluded for the analyses. All cases initially identified of stroke will be further validated by checking hospital medical records, and each case will be classified as related or nonrelated with a prior episode of CAP.

**D1. CAP-related episode of stroke**	Defined as an episode of stroke ocurring within the first 30 days after the onset of CAP.

**D2. CAP-nonrelated episode of stroke**	Defined as an episode of stroke ocurring more than 30 days after an episode of CAP or in a patient without history of a prior episode of CAP.

**E. All-cause death**	It includes patients who died for any cause during the study period. Cases will be identified from primary care information system of each participating PCC.

**E1. Death from CAP**	Defined as a patient who died (in hospital or not) within the first 30-days after the onset of CAP.

**E2. Death from AMI**	Defined as a patient who died within hospital stay or within the first 30-days after the diagnosis of AMI.

**E3. Death from stroke**	Defined as a patient who died within hospital stay or within the first 30 days after the onset of the stroke.

* All cases of CAP (hospitalised and outpatient) must be radiographically confirmed and validated by checking the clinical record with the use of a standardized data-collection instrument. CAP will be considered if, on conclusion of the medical record review, the physician reviewer verified this diagnosis and it was not a readmission, nosocomial pneumonia or another diagnosis.

** Pneumococcal CAP will initially be identified on the basis of ICD-9-CM codes for pneumococcal bacteraemia (codes 038.0, 038.2, 041.0, 041.2) or pneumococcal pneumonia (code 481) in the Hospitals and Emergency discharge diagnosis databases and ICD-10 code for pneumococcal pneumonia (code J13) in the Primary Care visits diagnosis databases. Laboratory records will also be used to identify cases of pneumococcal infections not detected in ICD-9-CM or ICD-10 discharge codes.

The occurrence of CAP will be established on the basis of an acute respiratory illness, with evidence of the presence of a new infiltrate in a chest radiograph.

Outpatient CAP will be identified from primary care or emergency visit (not hospitalised) with a code registered for pneumonia in the PCCs diagnoses databases (ICD-10 codes: J10.9, J11.9, J12 to J18) or in Emergency discharge databases (ICD-9-CM codes: 480 to 487.0). Hospitalisations for CAP will be identified on the basis of the listed primary diagnosis codes in the hospital discharge databases (ICD-9-CM codes 480 to 487.0).

All cases of CAP (hospitalised and outpatient) must be radiographically confirmed and validated by checking the clinical record with the use of a standardized data-collection instrument (which includes sociodemographical, clinical, exploratory, analytical and radiographical data at the time of diagnosis). CAP will be considered if, on conclusion of the medical record review, the physician reviewer verified this diagnosis and it was not a readmission, a nosocomial pneumonia or another diagnosis. Pneumonia severity index (PSI) and the simpler severity rule CURB-65 will be calculated according to criteria described in classical studies [38,39].

Hospitalisation for AMI will be initially identified on the basis of listed primary and secondary diagnosis codes in the reference hospital discharge database (ICD-9-CM code: 410).

Hospitalisation for stroke will also be initially identified on the basis of the listed primary and secondary diagnosis codes in hospital discharge databases (ICD-9-CM codes 430 to 437). Ischaemic strokes (codes 433, 434, 436 or 437) and hemorrhagic strokes (codes 430 to 432) will be considered, but transient ischemic attacks (code 435) will be excluded for the analyses.

All cases of AMI and stroke will be further validated by checking hospital medical records with a standardized data-collection instrument that includes clinical characteristics of the events, and they will be classified as "CAP-related" (occurring within 30 days after onset of pneumonia) or "CAP-nonrelated" (ocurring more than 30 days after an episode of pneumonia or ocurring among patients who have not suffered a previous CAP).

Because of the size of the study, an active following of study participants is not feasible. Nevertheless, the nine PCCs and two reference hospitals provide daily care to the inhabitants living in the selected study area, and they basically apply similar diagnostic checklist and treatment for patients presenting with a clinical suspicion of pneumonia, myocardial infarction or cerebrovascular accident.

### Vaccination history

Vaccination status of cohort members will be determined by a review of the PCCs' electronic clinical records, which contain specially designated fields for pneumococcal and influenza vaccinations. We assume that information in clinical records is complete, so a subject will be considered as unvaccinated when a vaccination was not recorded in the PCC clinical record.

Pneumococcal vaccination status (including number of dose of PPV-23 received in prior 10 years) will be determined at study start and it will be reevaluated at the end of the first and second year of the study period. Similarly, influenza vaccination status will be determined at the beginning of the study (according to reception or not of a dose of flu vaccine in prior autumn) and it will also be reevaluated every year of the study period.

### Covariates

Covariates will be age, sex, frequency of attendance (number of outpatient visits per year), history of pneumonia in 24-month before study start, history of coronary artery disease, history of prior stroke, presence of major cardiovascular risk factors (hypertension, hypercholesterolaemia, smoking, diabetes mellitus or obesity), presence of major comorbidities (chronic heart disease, chronic pulmonary disease, severe liver disease, chronic nephropathy or cancer) and immunological status. Table 2 lists covariates and shows criteria used to measure and capture underlying conditions and comorbidities in the study cohort.

**Table 2:** Definitions and criteria used to collect respiratory and cardiovascular risk factors and main comorbidities among cohort members in the CAPAMIS Study.*

UNDERLYING CONDITIONS	DEFINITIONS
**History of prior pneumonia**	Includes influenza-related pneumonia (J10.0 and J11.0), viral pneumonia (J12), pneumococcal pneumonia (J13), pneumonia due to Haemophillus influenzae (J14), bacterial pneumonia (J15), pneumonia due to other infectious organisms (J16), pneumonia in diseases classified elsewhere (J17) and pneumonia due to unspecified microorganisms (J18).

**History of coronary artery disease**	Includes angina (I20), acute myocardial infarction (I21), subsequent or recurrent myocardial infarction (I22) and chronic ischaemic heart disease (I25).

**History of stroke**	Includes haemorrhagic stroke (I60 to I62), cerebral infarction (I63) and stroke not specified as haemorrhage or infarction (I64); it does not include transient ischaemic attacks (code G45).

**Hypertension**	Defined as the presence of any code I10, I11, I12, I13 or I15.

**Hypercholesterolemia**	Defined as the presence of code E78.

**Diabetes mellitus**	Codes E10 to E14.

**Obesity**	Patients with registered code E66 (BMI>30%).

**Smoking**	Includes patients with current smoking (code F17 active at study start).

**Alcoholism**	Includes patients with code F10 active at study start.

**History of heart disease**	Includes congestive heart failure (I50), hypertensive heart disease (I11), cardiomyopathy (I42), cardiac dilatation or ventricular hypertrophy (I11.9 or I51.7) and coronary artery disease (I20, I21, I22 and I25).

**History of vascular ischaemia**	Besides coronary artery disease and stroke, it Includes atheroesclerosis (I70), acute arterial embolism or thrombosis (I74) and peripheric arterial disease or intermittent claudication (I73.9).

**Chronic pulmonary disease**	Includes chronic bronchitis or emphysema (J42 to J44) and asthma: (J45).

**Severe liver disease**	Includes chronic viral hepatitis (B18), alcoholic hepatitis (K70) and cirrhosis (K74).

**Severe nephropathy**	Includes nephrotic syndrome (N04 or N39.1), renal failure (N18, N19) and kidney dialysis (Y84.1) or transplantation.

**Immunocompromise**	Defined as the presence of any immunodeficiency (D80 to D84), AIDS (B20 to B24), anatomic or functional asplenia (D73.0 and Q89.0), cancer, severe nephropaty, or long-term corticosteroid therapy (20 mg/day of prednisone or equivalent).

* Data will be considered at the beginning of the study, and it will be defined according to the International Classification of Diseases 10th Revision (ICD-10) codes registered in the primary care clinical record of each cohort member at the start of the study.

### Statistical analysis

Absolute numbers, means and percentages will be computed to describe the patient population. The differences between groups will be evaluated using the chi-squared or Fisher's test for categorical variables, and the two tailed Student's test or ANOVA for continuous variables as appropriate.

The crude association between the outcomes and vaccination will be evaluated using incidence rates among vaccinated and unvaccinated subjects. The incidence of each event will be calculated as person-year considering in the denominator the sum of person-time contributed to each individual during study period.

Multivariable Cox proportional hazards models with time-varying covariates will be used to calculate hazards ratio (HR) and estimate the association between having received the pneumococcal vaccine and the time of the first outcome event during the study period. Repeated episodes will not be included in the analyses. Vaccine effectiveness will be estimated as (1 - HR) × 100.

Pneumococcal vaccination status will be a time-varying condition (e.g., individuals vaccinated after the beginning of the study) and persons will be considered to be vaccinated 14 days after vaccine administration. Annual influenza vaccine status will also be a time-varying covariate, whereas the other covariates will be defined at study start.

It will be checked for confounders, interactions and multicollinearity among the independent variables. The final models will be adjusted by all significant variables, as well as confounders and other baseline covariables judged of having clinical importance. The proportional hazards assumptions will be assessed adding the covariate by log-time interactions to the model and plotting the scaled and smoothed Schoenfeld residuals obtained from main effects model was possible [40]. Statistical significance will be set at p < 0.05 (two-tailed). The analyses will be performed using Stata/SE Version 9.1. (Stata Corp.)

### Ethical concerns

The study protocol has received ethics approval by the ethical committee of clinical research of the IDIAP Jordi Gol Foundation of the *Catalonian Health Institute *(ID P09/49) and it will be conducted in accordance with the general principles for observational studies.

The study protocol has received ethics approval by the ethical committee of *Catalonian Health Institute *(ID: CEIC IDIAP J. Gol P09/49) and it will be conducted in accordance with the general principles for observational studies. Given this is a non-interventional study, an informed consent for all 27000 study participants will not be required, but consent will be obtained to rewiev clinical records of those cohort members who suffer any study event during follow-up.

## Discussion

The clinical effectiveness of the PPV-23 is controversial, especially in preventing some outcomes such as CAP or AMI. However, given the demonstrated effectiveness of the vaccine in protecting individuals against IPD, commencing new randomised clinical trials in populations at risk where vaccine effectiveness and disease burden is known would create ethical difficulties. Thus, cohort studies are an acceptable alternative to estimate vaccine effectiveness against different pneumococcal-related outcomes among different populations at risk.

To our knowledge, this population-based cohort study in adults aged 60 years or older is the first large prospective study planed to evaluate the possible clinical effectiveness of pneumococcal vaccination against acute myocardial infarction and stroke, related or not with prior episodes of pneumonia.

The results of the study will contribute to clarify the controversial effect of the PPV-23 in preventing community-acquired pneumonia and they will be important to determine the possible role of pneumococcal vaccination in cardiovascular prevention.

This study has been conceived and designed to investigate the clinical effectiveness of the PPV-23 against pneumonia and acute cardiovascular events. Nevertheless, in addition to the primary objectives of the study, several substudy objectives are currently under development to maximise opportunities to learn about the health conditions in this older adult population-based cohort.

## Competing interests

The authors declare that they have no competing interests.

## Authors' contributions

AVC wrote the research proposal which was approved by the *Instituto de Salud Carlos III *for funding; I. Hospital, CD, ES, XR and CMF conducted data collection; AVC and OO supervised the data collection; AVC wrote the draft of the manuscript; all authors read and approved the final manuscript.

## Pre-publication history

The pre-publication history for this paper can be accessed here:

http://www.biomedcentral.com/1471-2458/10/25/prepub
